# Backers investment behavior on explicit and implicit factors in reward-based crowdfunding based on ELM theory

**DOI:** 10.1371/journal.pone.0236979

**Published:** 2020-08-06

**Authors:** Rui Hou, Leiming Li, Bingquan Liu

**Affiliations:** School of Economics & Management, China University of Petroleum (hua dong), Qingdao, P.R. China; Shandong University of Science and Technology, CHINA

## Abstract

The aim of this study is to identify the dynamic explicit and implicit information factors which displayed on the webpage of platforms that influence backers’ investment decision-making behavior. We analyze the connections among these factors by collecting the longitudinal dataset from reward-based crowdfunding platform. Based on ELM model, we establish Fixed Estimation Panel Data Model respectively according to explicit and implicit factors and take Funding Status (crowdfunding results) as the moderating variable to observe the goal gradient effect. Results indicate that most variables in the central route affect backers' investment behavior positively, while most variables in the periphery route have a negative impact on backers' investment behavior. The Funding Status has a significant negative moderating effect on the explicit variables, and has no significant moderating effect on the implicit information variables of the project. In addition, we upgrade the econometric method used by previous scholars, which could improve the accuracy of the FE model. Furthermore, we find strong support for the herding effect in reward-based crowdfunding and the intensity tends to decrease before the funding goal draws near.

## Introduction

As an important branch of crowdfunding, reward-based crowdfunding has attracted many scholars’ attention these years. An emerging literature on reward-based crowdfunding mainly focus on two aspects, one is the static factors driving a campaign's success. Such factors include both project-level signals, for instance project funding goal [1], project design [2], product categories [3] and other project preparedness [4, 5] and individual-level signals, such as creator’s gender [6], experience of creator [7] and social capital of creator [8]. The other one is the influence of backers' decision behavior on dynamic information factors. For example, Galak et al. [9] point out that besides the influence of traditional investment factors, the backer's emotion (sympathy, curiosity, etc.) towards the project or the creator is also one of the factors that will affect the backer's decision behavior. Some scholars focus on the importance of early contributions in crowdfunding as the herding behavior [8]. Kuppuswamy and Bayus [10], leveraging data from Kickstarter platforms, find that backer’s support increases as the goal end state approaches according to the Perceived Impact [11] and Goal Gradient Effect [12].

After an in-depth thinking in the dynamic decision behavior of backers, we come up with some different understandings, especially in data processing method and statistical approaches, which is the main research point in this paper. The following is a brief review of the prior researches of backers' investment decision behavior.

To our best known, most of the current research on backers’ dynamic information in reward-based crowdfunding are based on the econometric method proposed by Kuppuswamy and Bayus [10]. Although Kuppuswamy provides a common way for the following researchers, we think that there are still some problems in variables selection when construct the panel data model.

1. Selection of dependent variable.
  Kuppuswamy and Bayus [13], and the following researchers such as Chen et al. [14], Zhen et al. [15], they all use the increased backers per day as the dependent variable. It is improper to take this variable as the proxy variable of backers’ decision behavior. The reasons are, (1) Crowdfunding is a sort of financing and investment behavior, according to the needs of the tripartite subject in crowdfunding, creators need to raise money to start their project; Platform need to earn commissions or platform traffic from crowdfunding; Backers need to get novel products by investing the creators [1] and gain a sense of achievement and personal satisfaction by helping the creators to accomplish their projects under the pro-social behavior [9, 16]. In addition, the criterion for determining the success or failure of a project is also depending on the amount of raised money. Therefore, the research topic should be based on raised money. Considering the mechanism of crowdfunding, the cumulative funding rate [(money pledged) / (funding goal)]—the percentage of a funding goal—is a better choice for the dependent variable. Several previous studies have employed the rate-based measures on project performance [4, 17]. (2) To provide backers with a variety of supporting options, each project has multiple reward selection levels and different reward level usually has huge different funding gap. (3) Some platforms especially in China, adds “lottery draw” and “charity contribution” in the reward selection level, that will lead to serious disproportion between supporting backers and raised money of the projects. This phenomenon of increasing the number of backers but without increasing the amount of raised money will affect the regression results. From the above analyses, the increased backers are not comprehensive enough to explain the investment behavior of backers.
2. Selection of independent variables.
  In the prior studies, scholars employ first 7 days (first week) and last 7 days (last week), total 14 dummy variables as independent variables in data panel model [13, 15, 18]. In this way, the entire financing cycle is divided into three periods but the three intervals are unevenly distributed. Therefore, the regression result can only roughly show the change amid the periods between the beginning and the end periods. That will not give an overall observation of the entire crowdfunding duration.
  Besides, Kuppuswamy and Bayus [13] and Gu and Zhao [18] removed a large number of project samples to ensure the balance of the data panel. They just analyzed the projects of 30 days duration. There are 44% projects in Kickstarter choosing 30 days as crowdfunding duration [4]. That means more than half of data will be deleted. It will affect the accuracy of regression result.

## Theoretical background and hypothesis

### ELM in crowdfunding

The elaboration likelihood model (ELM) is a theory of persuasion developed by Richard Petty and John Cacioppo in the 1980 [19]. ELM denotes that the overall evaluation could be influenced via two distinct routes, which is the central route and peripheral route [20]. According to Petty and Cacioppo [20], the key concept is the idea of elaboration. At higher levels of elaboration, people are more likely to think over an issue carefully, but at lower levels, they may make decisions that are less carefully thought out.

In recent years, scholars have employed ELM in a variety of internet-based context. A body of evidence suggests that enterprises can use internet-based communications as an effective method for changing the purchase intentions of potential customers [21]. The ELM provides a better understanding that entrepreneurs attract potential customers by using the information about their products. Moreover, ELM is widely used in the products advertising [22]. Information of project webpage on the platform can be regarded as the advertisement of the crowdfunding project [23]. Therefore, we adopt the project webpage information as the persuasion to construct an ELM model. According the ELM, the central route involves factors that can provide evaluation of crowdfunding projects for potential backers, which are important determinants for potential backers to decide whether to invest or not. It can offer reference for backers' decision-making. On the other hand, the peripheral route is not a direct description of a project or product, it is commonly used in other indirect influencing factors.

This paper employs ELM to explain backers' investment decision-making behavior for two reasons. One is the whole crowdfunding project information presentation on the webpage can be regarded as the persuasion information that convince potential backers to join the project. The functions of a project's information exhibition page are fitted for the applicable conditions of ELM. The other one is backers need to process all the information they received before they make their investment decision, therefore the creators try hard to attract potential backers' attention. The process of reward-based crowdfunding projects conforms to the explanatory of ELM.

In the extant crowdfunding studies, Allison et al. [24] take the product quality and the background of the creator as the central route, and project description (semantic, emotional, etc.) as the peripheral route. Zheng et al. [25] adopt the experience of the creator (number of success or failure) as the central route, and the social relationship of the creator (number of crowdfunding project launches, interactive information, etc.) as the peripheral route. Zeng et al. [26] employ the information displayed by the project (funding goal, funding level, etc.) as the central route, and the activity of creators (project updates, project topics, etc.) as the peripheral route. Through the route of ELM, it can produce a better explain for different backers' access and processing information mode, which will help us know more about investment decision-making behavior of backers.

Based on the study of backers' investment decision-making behavior, according to ELM route selection by Allison et al. [24], we adopt the dynamic information of the project as the way to distinguish the route (central or peripheral) in the light of the backers' cognitive and affective. We will divide the dynamic information of the project based on the EML theory. In addition, the dynamic information is composed of explicit and implicit dynamic information.

The explicit information presented on the project webpage is the information that backers can perceive in real time. It can be divided into project progress information and mutual exchange information. Commonly in ELM, the central route delivers complex information and peripheral route transmits a less-effortful thought process of individuals' evaluations [27]. Therefore, in this paper, the project progress information is classified as the central route, and the interactive communication information is classified as the peripheral route. Previous scholars [23–26] mostly adopted the product quality of the project and creators’ ability as the central route. It is different from the extant research in our route selection. The reason for the difference is our research is focus on backers’ investment decision-making behavior which is based on the dynamic information including explicit and implicit of the project. But the existing researches are all aimed at static information. In addition, the fixed effect panel data regression model used in this paper can control the heterogeneity (invariant information) of the project itself. Besides, according to the EML theory, information in center route should be the essential information that will be carefully studied and affect the judgment of potential backers. Hence, information such as cumulative / increment funding of project (platform) and cumulative / increment backers of project (platform) dynamically displayed on the platform can proxy the nature of project and platform. The essence of crowdfunding project is financing and the size of platform traffic can reflect the quality of a platform. It is the first time that ELM model is applied into dynamic information of crowdfunding project.

**Fig 1:**
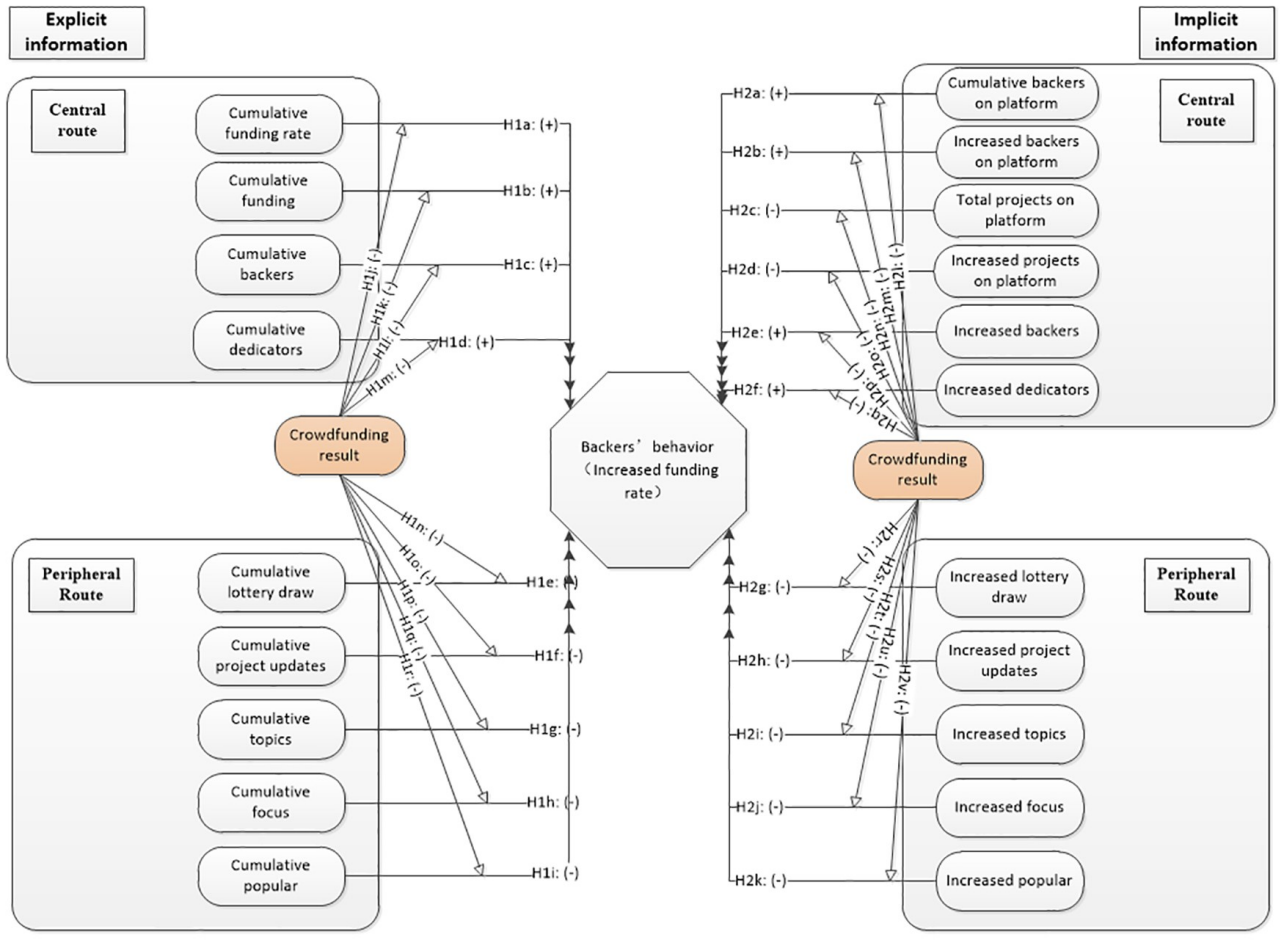
Project dynamic implicit/explicit factors frame based on ELM model.

Through the above analysis, we depict the explicit and implicit information framework of project below in Fig 1. We will develop and test the theoretical model in the following section.

### Hypothesis of the influence of project dynamic explicit and implicit information on backers' funding behavior

#### Hypothesis of central route on backers' funding behavior

All the variables used this section are defined in Table 1 in the section of 4.3.2.

**Table 1:** Variable definition (Normalization).

Variable		Logogram	Description	Instruction
**Individual Variable**	Number of observations	IndividualVar	Crowdfunding projects	visible
**Time Variable**	Normalization Days	TimeVarNor	Normalization days (1–20 days)	visible
**Dependent Variable**	Increased Funding Rate	PledgesRIncNorY	(cumulative funding of the current day—cumulative funding of the current day before) / funding goal	invisible
	Increased Funding	PledgesIncrNorYLn	Increased Financing on project	invisible
**Independent Variable**	Cumulative Funding Rate	PledgesRCumNor	Cumulative Funding Rate on each project per day	visible
	Cumulative Funding	PledgesCumNorLn	Cumulative Funding on each project per day	visible
	Increased Lottery Draw	DrawNumIncEachdNor	Increased Lottery Draw on each project per day	invisible
	Cumulative Lottery Draw	DrawNumCumEachdNor	Cumulative Lottery Draw on each project per day	visible
	Increased Dedicators	DediNumIncEachdNor	Cumulative Lottery Draw on each project per day	invisible
	Cumulative Dedicators	DediNumCumEachdNor	Cumulative Dedicators on each project per day	visible
	Increased Backers	BackersIncEachdNorLn	Increased Backers on each project per day	invisible
	Cumulative Backers	BackersCumEachdNorLn	Cumulative Backers on each project per day	visible
	Increased Focus	FocusIncEachdNor	Increased Focus on each project per day	invisible
	Cumulative Focus	FocusCumEachdNor	Cumulative Focus on each project per day	visible
	Increased popular	NiceIncEachdNor	Increased popular on each project per day	invisible
	Cumulative popular	NiceCumEachdNor	Cumulative popular on each project per day	visible
	Increased Focus and Popular	FocusNiceIncNorLn	Increased Focus and Popular on each project per day	invisible
	Cumulative Focus and Popular	FocusNiceCumNorLn	Cumulative Focus and Popular on each project per day	visible
	Increased project updates	ProgressIncEachdNor	Increased project updates on each project per day	invisible
	Cumulative project updates	ProgressCumEachdNor	Cumulative project updates on each project per day	visible
	Increased Topics	TopicIncEachdNor	Increased Topics on each project per day	invisible
	Cumulative Topics	TopicCumEachdNor	Cumulative Topics on each project per day	visible
	Total Projects on Platform	ActiProjNumEachdNorLn	The total number of active projects on platform each day	invisible
	Increased Projects on Platform	ActiProjNumIncEachdNorLn	The Increased number of active projects on platform each day	invisible
	Total Funding on Platform	ActiProjFundsEachdNor	The total financing of active projects on platform each day	invisible
	Increased Funding on Platform	ActiProjFundsIncEachdNor	The increased financing of active projects on platform each day	invisible
	Increased Backers on Platform	AllBackersIncEachdNorLn	The increased number of backers on platform each day	invisible
	Cumulative Backers on Platform	AllBackersICumEachdNorLn	The cumulative number of backers on platform each day	invisible
	Crowdfunding Result	IsSuccEachNor	Crowdfunding status (dummy variable: success = 1; failure = 0)	invisible

1. Explicit information hypothesis
  For the central route of explicit information, it includes four factors: Cumulative funding rate, Cumulative funding, Cumulative backers and Cumulative dedicators. From the subjective feelings of backers, the increase of the Cumulative funding rate, the Cumulative funding and the Cumulative backers represent project quality that is accepted by most backers. And the Cumulative dedicators are the donation number of backers, which does not belong to interactive information. The more dedicators the more pledged, so that the number of dedicators is also an indicator of project quality. Therefore, the above four factors should have a significant and positive impact on the decision-making behavior of potential backers.
  In the extant research, Burtch et al. [28] pointed out that backers tend to pay attention to other backers’ investment situation on the same project before they make decision. Ye and Du [29] studied the decision behavior of backers through the hoteling model of two-sided markets and concluded that the attractiveness of the project to potential backers was significantly and positively correlated with the number of participating backers in the project. Furthermore, many scholars found the existence of herding effect in crowdfunding [8]. Yum et al. [30] believed that when there is information asymmetry in the crowdfunding market, backers will take the projects with more funding amount as the signal of higher product quality, which will lead to herding behavior. Under herding effect, the above cumulative factors should have a positive impact on backers' decision-making behavior. According to the above analysis, we propose the following research hypothesis:
  H1a: The cumulative funding rate will positively affect the decision-making behavior of backers.
  H1b: The cumulative funding will have a positive relationship with decision-making behavior of backers.
  H1c: The cumulative backers will positively affect the decision-making behavior of backers.
  H1d: The cumulative dedicators will positively affect the decision-making behavior of backers.
2. Implicit information hypothesis
  For the central route of implicit information, it includes six factors: Total projects on platform, Increased projects on platform, Cumulative backers on platform, Increased backers on platform, Increased backers, Increased dedicators. Because of the multiple collinearities, two factors of “total funding amount of platform” and “increased funding amount of platform” are not listed. Please refer to the following section.
  Except for “Increased backers” and “Increased dedicators” on project level can be obtained by carefully observing the next day's project data changing, the first four implicit information factors are on the platform level, which cannot be observed by normal potential backers. These kind of information needs special data retrieval method, such as web crawling software, etc. The data retrieval approach is in line with the definition of central route in ELM theory, which requires the information receiver to make more cognitive efforts and it is not easily to get [31]. As a result, this paper takes the above implicit factors as the central route.
  Moreover, the factors of “Cumulative backers on platform” and “Increased backers on platform” are both related to the actual number of supporting backers. Therefore, the impact of these two factors on the decision-making behavior of potential backers should be positive, especially with herding effect. For factors of “Total projects on platform” and “Increased projects on platform”, due to the “Kickstarter fatigue” as proposed by some industry person, in which potential backers are becoming weary from the increasing number of projects asking for their financial contributions [32]. Besides, the increase of the total number of platform projects will inevitably disperse the investment choice of limited potential backers. So these two factors should be negative to the decision-making behavior of potential backers. For the factors of "Increased backers" and "Increased dedicators" on project level, although they cannot be easily perceived by other potential backers, the increase of these two factors means the spike of funding amount, which has a direct impact on the dependent variable. It's completely opposite to the number of "lottery draw”, “popular” and “focus”. They are more of a kind of bystander nature. Such different measurement between these factors will lead to different effects on the increased funding rate (dependent variable). This is in line with the previous studies using panel data [33, 34]. With the cumulative numbers grow, the herding effect will be triggered, then “Increased backers” and “Increased dedicators” factors will be positive impact on decision-making behavior of potential backers. According to the above analysis, we propose the following research hypothesis:
  H2a: The cumulative backers on platform will positively affect decision-making behavior of potential backers.
  H2b: The increased backers on platform will positively affect decision-making behavior of potential backers.
  H2c: The total projects on platform will negatively affect decision-making behavior of potential backers.
  H2d: The increased projects on platform will negatively affect decision-making behavior of potential backers.
  H2e: The increased backers will positively affect decision-making behavior of potential backers.
  H2f: The increased dedicators will positively affect decision-making behavior of potential backers.

#### Hypothesis of peripheral route on backers' funding behavior

For the peripheral route of explicit information, it includes four factors: Cumulative lottery draw, Cumulative focus and popular, Cumulative project updates, Cumulative topics. As the number of focus and popular are highly correlated, so we combined the two variables into one, which will be described in the following chapters.

For the peripheral route of implicit information, it includes five factors: Increased lottery draw, Increased focus, Increased popular, Increased project updates, Increased topics.

These factors are interactive information between creators and backers and will not directly affect the funding of the project. Although more interaction between the creators and backers means more attention of the project and higher popularity with the backers. It should have a positive impact on the decision-making behavior of potential backers. In addition, some scholars [35, 36] use cross-sectional data empirically conclude that the interaction of backers and creators have a positive impact on project funding performance.

But other scholars have different opinion, for instance, through data envelopment analysis (DEA) and panel model, Wang et al. [37] empirically concluded that the number of topics and the number of potential backers who concerned about crowdfunding projects had a significant and negative impact on project funding efficiency. In this paper, we think that the negative effect is more consistent with the actual condition under the dynamic longitudinal data. The reasons are as follows: First, for real-time interactive information between creator and backer, with the development of crowdfunding process, it means that the project information disclosed by interaction will gradually increase. Therefore, as the number of participants increased, it will trigger the bystander effect and reduce the support by potential backers. Second, when the project information provided by the creator is insufficient or the explanation of which the backers’ enquiry is not detailed or satisfied, that will surely reduce the trust of the potential backers. Third, there are positive and negative comments display on the project webpage. When the negative comments more than the positive ones, the bad comments will decrease the potential backers’ funding willing. Based on the above points of view, this paper holds that all interactive information (i.e., explicit and implicit information in peripheral route) should have a negative impact on the project performance (backers' funding behavior). Formally, we hypothesize:

1. Explicit information of peripheral route hypothesis
  H1e: The cumulative lottery draw will negatively affect decision-making behavior of potential backers.
  H1f: The cumulative project updates will negatively affect decision-making behavior of potential backers.
  H1g: The cumulative topics will negatively affect decision-making behavior of potential backers.
  H1h: The cumulative focus will negatively affect decision-making behavior of potential backers.
  H1i: The cumulative popular will negatively affect decision-making behavior of potential backers.
2. Implicit information of peripheral route hypothesis
  H2g: The increased lottery draw will negatively affect decision-making behavior of potential backers.
  H2h: The increased project updates will negatively affect decision-making behavior of potential backers.
  H2i: The increased topics will negatively affect decision-making behavior of potential backers.
  H2j: The increased focus will negatively affect decision-making behavior of potential backers.
  H2k: The increased popular will negatively affect decision-making behavior of potential backers.

#### The moderating roles of funded status on backers’ decision-making behavior

Currently, there are few studies on moderating effect of dynamic variables of crowdfunding projects. But Wang et al. [33] considered that the decision-making behavior of backers was moderated by different variables. The Funded status is a real-time state of the ongoing project. When the current funding is not reaching the financing target goal, the project is in a non-funded status, otherwise the project is in a funded status.

Some crowdfunding projects have gained more backers' favor because their heterogeneity, so they have accomplished their target goal before the deadline. In the state of funded status, because the project has succeeded, backers can obtain crowdfunding products if they support. For potential backers, it reduces the uncertainty of the project to a certain extent, even affecting the investment decisions. That means the funded status will moderate the variables in central and peripheral route and affect the funding performance of the project. Therefore, the influence of explicit and implicit factors on potential backers’ investment decision-making behavior will depend on the funded status. As the goal gradient effect exists in reward-based crowdfunding [10, 18], this paper believes that once the project reaches its funding goal, the subsequent funding performance will be influenced by the goal gradient effect. And the funded status will have a negative impact on all dynamic factors, moderating the impact on the investment behavior of potential backers. Thus, we propose the following hypothesis.

1. The moderating roles in explicit factors
  H1j: Funded status (crowdfunding results) negatively moderates the effect of cumulative funding rate on potential backers’ decision-making behavior.
  H1k: Funded status (crowdfunding results) negatively moderates the effect of cumulative funding on potential backers’ decision-making behavior.
  H1l: Funded status (crowdfunding results) negatively moderates the effect of cumulative backers on potential backers’ decision-making behavior.
  H1m: Funded status (crowdfunding results) negatively moderates the effect of cumulative dedicators on potential backers’ decision-making behavior.
  H1n: Funded status (crowdfunding results) negatively moderates the effect of cumulative lottery draw on potential backers’ decision-making behavior.
  H1o: Funded status (crowdfunding results) negatively moderates the effect of cumulative project updates on potential backers’ decision-making behavior.
  H1p: Funded status (crowdfunding results) negatively moderates the effect of cumulative topics on potential backers’ decision-making behavior.
  H1q: Funded status (crowdfunding results) negatively moderates the effect of cumulative focus on potential backers’ decision-making behavior.
  H1r: Funded status (crowdfunding results) negatively moderates the effect of cumulative popular on potential backers’ decision-making behavior.
2. The moderating roles in implicit factors
  H2l: Funded status (crowdfunding results) negatively moderates the effect of platform cumulative backers on potential backers’ decision-making behavior.
  H2m: Funded status (crowdfunding results) negatively moderates the effect of platform increased backers on potential backers’ decision-making behavior.
  H2n: Funded status (crowdfunding results) negatively moderates the effect of total platform projects on potential backers’ decision-making behavior.
  H2o: Funded status (crowdfunding results) negatively moderates the effect of increased platform projects on potential backers’ decision-making behavior.
  H2p: Funded status (crowdfunding results) negatively moderates the effect of increased backers on potential backers’ decision-making behavior.
  H2q: Funded status (crowdfunding results) negatively moderates the effect of increased dedicators on potential backers’ decision-making behavior.
  H2r: Funded status (crowdfunding results) negatively moderates the effect of increased lottery draw on potential backers’ decision-making behavior.
  H2s: Funded status (crowdfunding results) negatively moderates the effect of increased updates on potential backers’ decision-making behavior.
  H2t: Funded status (crowdfunding results) negatively moderates the effect of increased topics on potential backers’ decision-making behavior.
  H2u: Funded status (crowdfunding results) negatively moderates the effect of increased focus on potential backers’ decision-making behavior.
  H2v: Funded status (crowdfunding results) negatively moderates the effect of increased popular on potential backers’ decision-making behavior.

## Research method

### Data sources

Since Kickstarter platform is widely studied by the prior scholars [4, 10], we focus on the JD reward-based crowdfunding platform in China. JD has been cited as the largest reward-based crowdfunding platform in China, in terms of both the number of projects and the level of capital pledged [26]. To capture a cross-section of all crowdfunding projects on the platform, we wrote a computer program using Visual Basic(VB) and Win-http protocol to extract information about all projects posted on the platform, collecting all project pages and adding new projects from 16:00 p.m. to 19:00 p.m. every day, over a 105-day period from October 7, 2019 to January 20, 2020. This resulted in a data set on 927 projects. According to the data selecting method (refer to section 4.3), 64 projects with funding duration less than or equal to 20 days and 4 canceled projects during the funding period are eliminated. Therefore, there are 859 projects, 28878 panel data set in total. On average, the creators in the data set pledged accounting for 55.71% of their stated funding goals. The success rate is little higher than Kickstarter, on which the success rate is approximately 48% [4].

The average funding goal of the sample is ¥48477.26, and the median is Y30000; The average funding duration is 33.72 days and the median is 30 days; The daily increased funding rate of the project is 4.24%, that is to say, all projects achieve 4.24% of their funding goal on average in each funding day. In 470 successful projects, the median financing amount is ¥30000, with an average value of ¥38669.5. The median of actual funding is ¥51879.3, with an average of ¥75246.1. The median project duration is 30 days, with the average of 34.4 days.

### Summary statistics

This section focuses on a descriptive statistical analysis of the data set involved in this study, including descriptive analysis of normalized data, distribution figure and the original data analysis.

#### Statistical analysis after normalization

Summaries of this data after normalization can be found in Table 2, in which the normalization data will be higher than that of the actual funding data daily, because we have compressed actual funding days. The following is a brief analysis of the normalization data according to Table 2. Each day means each normalization day.

1. The average increased funding rate daily of each project is 7.1%.
2. The average increased pledges daily is ¥2192.
3. An average of 9.5 backers supported per project per day.
4. The number of lottery draw and dedicators increased by 0.15 and 1.64 per day on average, indicating that the potential backers are not motivated to participate in the two items. This may be the reason why Kickstarter and other reward-based crowdfunding platform do not set up these two items.
5. About 621 new projects launched every day, with a funding amount of ¥0.75 million and 2092 supporting backers will be added each day.
6. About 32% population of projects are in funded status.
7. Most Min values of Increased variables are negative, that because the Increased value equals today's value minus the previous day's value, if previous day’s value is greater than today’s value then it will result negative values. But negative values are not possible for cumulative variables.

**Table 2:** Summary statistic of normalized variables.

Variable	Obs	Mean	Std. Dev.	Min	Max
PledgesRIncNorY	17180	0.0712317	0.3760353	-15.34	23.22
PledgesRCumNor	17180	0.8562142	1.741463	0	40.1
PledgesCumNor	17180	29457.11	101860.3	1	4010461
PledgesIncrNorY	17180	2191.627	24791.12	-15762	2364016
BackersIncEachdNor	17180	9.520256	80.943	-5165	6741
BackersCumEachdNor	17180	119.7024	331.0507	0	8135
ActiProjNumEachdNor	17180	621.5742	302.8763	5	1909
ActiProjNumIncEachdNor	17180	-0.528114	21.63549	-59	111
ActiProjFundsEachdNor	17180	19100000	9537480	110484	68500000
ActiProjFundsIncEachdNor	17180	751291.5	541809.1	-124800	4357670
AllBackersIncEachdNor	17180	2092.73	1461.5	-6700	9597
AllBackersICumEachdNor	17180	46684.29	17132.51	414	72813
DrawNumIncEachdNor	17180	0.151688	4.951883	-1	361
DrawNumCumEachdNor	17180	1.870489	29.86387	0	890
DediNumIncEachdNor	17180	1.642899	27.44699	-1046	1231
DediNumCumEachdNor	17180	17.88981	135.1254	0	2189
FocusIncEachdNor	17180	18.87247	195.8762	-31	18320
FocusCumEachdNor	17180	329.2437	1152.817	0	31909
NiceIncEachdNor	17180	15.64971	180.6317	0	15360
NiceCumEachdNor	17180	286.5086	1061.889	0	28974
ProgressIncEachdNor	17180	0.2157742	0.6937106	-10	11
ProgressCumEachdNor	17180	2.728754	4.361629	0	35
TopicIncEachdNor	17180	1.602619	5.847381	-95	112
TopicCumEachdNor	17180	22.30605	30.55915	0	284
IsSuccEachNor	17180	0.3218859	0.4672131	0	1

#### Description and figure of original data set

**Fig 2:**
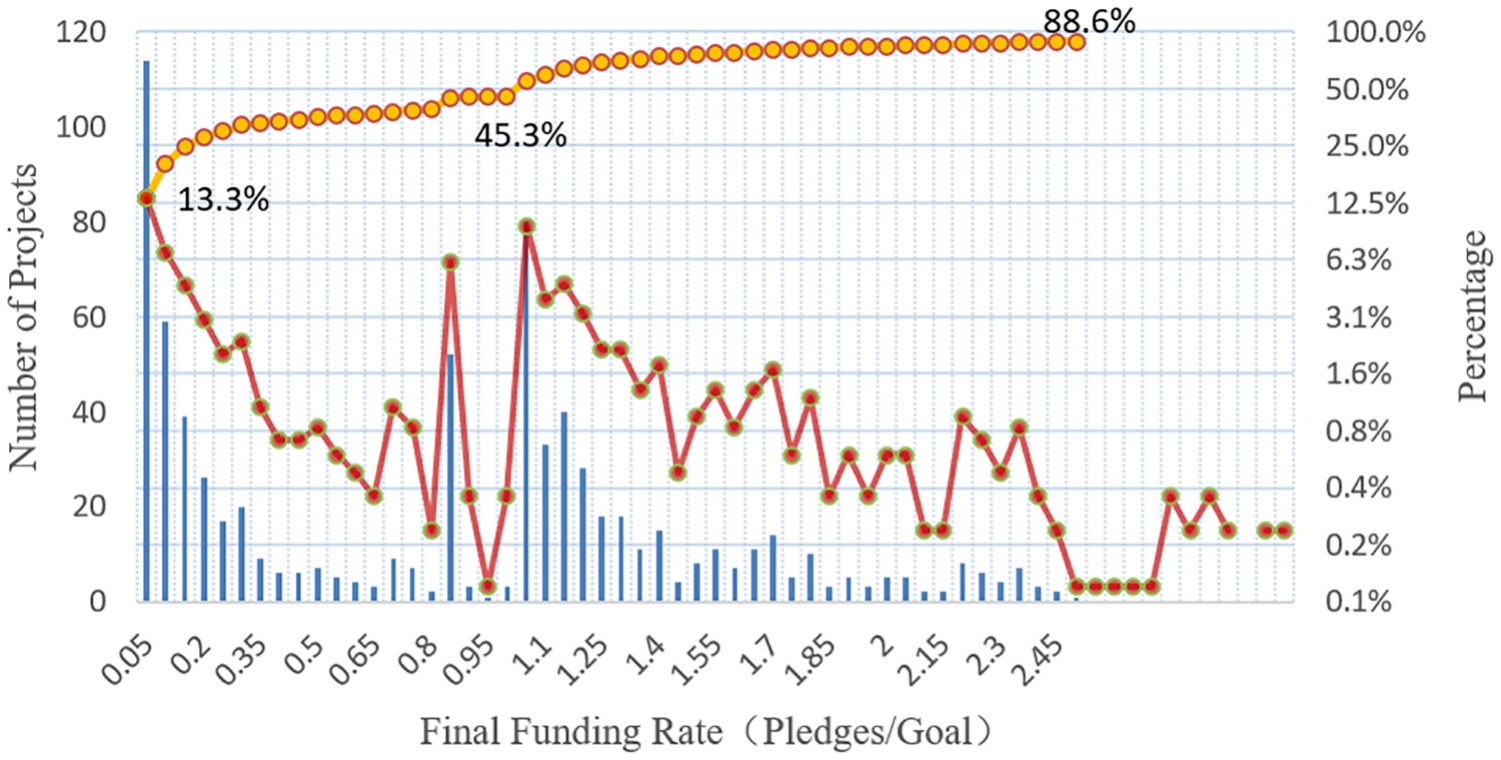
Distribution of project funding rate (logarithmic axis).

In order to show the characteristics of the original data set, especially the distribution of the project final funding, this paper chooses the final funding rate of the project as the horizontal axis, the number of projects (left) and the proportion of projects (right) as the vertical axis, and draws the project distribution of funding rate (Fig 2). From the figure, we can find that 13.3% of the projects cannot reach 5% of their funding goal and 9.3% of the projects finish at 105% of their funding goal. Nearly 90% of the projects will not finance more than 2.5 times of their funding goal, that means setting a reasonable funding goal is crucial for the campaigns. Moreover, 6.1% of the projects are terminated at 85% of their funding goal. A great possibility of this phenomenon is that when the project funding rate is near 80%, the creator himself or his relatives and friends will “self-funding”, hoping to intrigue herding effect and perceived impact of potential backers before the end of the project [10, 38]. Apparently, the effect of “self-funding” around the 80% is not ideal, most of the creators give up the action after reaching a certain proportion of “self-funding” (about 5%). In this case, the rate of the final funding generally stopped at about 85%. As the platform fee of JD has reached 10% of the total financing amount. This cost is also a consideration for the creators that is consistent with the research of Cumming et al. [39].

### Variables selection and correlation test

The purpose of this paper is to explore the backers’ investment behavior with dynamic explicit and implicit information factors. Based on the hypotheses proposed in section 3.2, this section will define the variables involved in the study in detail and give the measurement criteria and calculation methods of the variables. Finally, the correlation matrix between the variables is given.

#### Variables selection

1. Individual variable: 859 projects.
2. Time variable: Normalization duration (1–20 days).
3. Dependent variable: Increased Funding Rate (PledgesRIncY).
  *Increased funding rate (PledgesRIncY)*. Proportion of daily increased financing amount of project funding goal. It is an explanation for the changing of backers' behavior.
  *Calculation method*. (cumulative funding of the current day—cumulative funding of the current day before) / funding goal.
  Based on the following reasons, we adopt Increased Funding Rate instead of Increased Funding or Increased backers as dependent variable. First, each project has its different funding goal and crowdfunding takes the funding goal as the criteria of project success or failure. Neither of increased financing amount or supporting backers could show the impact crowdfunding results. But the Increased Funding Rate has the influence of the crowdfunding results, 100% means the creator achieved the target goal. In addition, platform display the current funding rate in a significant place on the webpage, which will inevitably impact on the decision-making behavior of potential backers when they browse the webpage of the project. Second, the existing empirical research have come to a conclusion that the funding goal has an important influence on the project result and backers' decision-making behavior [4, 40]; Third, for each project, funding goal is unchanged during the funding period, so it cannot be adopted as a variable in the Fixed effect panel data model regression, but Increased funding rate can.
  Combining the above reasons, including the funding goal in dependent variable not only makes the factor carry more explanatory information, but also makes the regression model closer to the description of backers' behavior. In addition, most of the crowdfunding platforms apply all-or-nothing model which established by Kickstarter in 2009 as a measure to protect creators, and to minimize risk for everyone. By not releasing funds unless a project meets its goal, this ensures that creators have enough money to do what they promised and they’re not expected to complete a project without the funds necessary to do so. This also assures backers that they’re only funding creative ideas that are set to succeed. That is the essence of crowdfunding and the funding goal is the only factor to decide all or nothing which the creator can get. Therefore, in this study, we believe that the Increased funding rate is more suitable as a dependent variable than others. Since the Cumulative funding can also explain the backer's investment behavior in a certain extent, this paper will use it as the dependent variable in the subsequent robustness test.
4. Independent variables
  - Explicit dynamic factors
    There are 9 dynamic factors displayed on the project webpage, including “Cumulative funding rate”, “Cumulative funding”, “Cumulative backers”, “Cumulative dedicators”, “Cumulative lottery draw”, “Cumulative project updates”, “Cumulative topics”, “Cumulative focus”, “Cumulative popular”. Among these factors “Cumulative focus” and “Cumulative popular” are highly correlated (Table 3). Therefore, the average of the two factors is used as the new variable “Cumulative focus and popular”. These cumulative factors update on the project page every day, but how the dynamic change of these factors will affect the backers’ investment behavior is exactly what we propose to find out. Therefore, the above eight factors are used as independent variables of Eq 1, and take the variable of “crowdfunding results” as moderating variable.
  - **Implicit dynamic factors**
    Some of the information on the reward-based crowdfunding platform is invisible to backers. Such as “Total projects on platform”, “Increased projects on platform”, “Increased backers on platform”, “Cumulative backers on platform”, “Total funding on platform”, “Increased funding on platform”, “Increased lottery draw”, “Increased dedicators”, “Increased backers”, “Increased focus and popular”, “Increased project updates”, “Increased topics”, 12 factors. Because of the multiple collinearities, “Total funding on platform” and “Increased funding on platform” will delete. The “crowdfunding results” is also used as the moderating variable for each independent variable.

**Table 3:** Correlation matrix of dependent variables.

	变量	1	2	3	4	5	6	7	8	9	10	11	12	13	14	15	16	17	18	19	20	21	22	23
1	PledgesRCumNor	1																						
2	IsSuccEachNor	.528`	1																					
3	PledgesCumNorLn	.439`	.583`	1																				
4	ActiProjNumEachdNorLn	.052`	.025`	.039`	1																			
5	ActiProjNumIncEachdNorLn	-.069`	-.071`	-.030`	-.017**	1																		
6	ActiProjFundsEachdNorLn	.051`	.040`	.053`	.959`	0.004	1																	
7	ActiProjFundsIncEachdNorLn	.008	.001	-.001	.245`	-.031`	.213`	1																
8	AllBackersIncEachdNorLn	-.002	-.014*	-.025`	.293`	.049`	.253`	.767`	1															
9	AllBackersICumEachdNorLn	.040`	.031`	.007	.556`	-.128`	.787`	.207`	.361`	1														
10	DrawNumIncEachdNor	.031`	.041`	.037`	.028`	.019**	.034`	.01	.01	.016**	1													
11	DrawNumCumEachdNor	.104`	.085`	.085`	.043`	-.018**	.043`	.012	0.008	.022`	.343`	1												
12	DediNumIncEachdNor	.099`	.033`	.031`	.024`	.002	.024`	.008	.015**	.018**	.007	.004	1											
13	DediNumCumEachdNor	.120`	.037`	.069`	.032`	-0.01	.030`	.012	.020`	.048`	.022`	.030`	.353`	1										
14	BackersIncEachdNorLn	.333`	.282`	.354`	.113`	.070`	.086`	.065`	.035`	-.031`	.098`	.105`	.222`	.120`	1									
15	BackersCumEachdNorLn	.495`	.572`	.806`	.049`	-.045`	.059`	0.003	-.013*	.048`	.058`	.129`	.108`	.250`	.510`	1								
16	FocusIncEachdNorLn	.181`	.152`	.256`	.125`	.113`	.096`	.043`	0	-.094`	.061`	.087`	.060`	.020`	.563`	.274`	1							
17	FocusCumEachdNorLn	.204`	.249`	.436`	-.017**	.005	.017**	-.023`	-.044`	0.001	.042`	.095`	.015*	.070`	.163`	.444`	.296`	1						
18	NiceIncEachdNorLn	.051`	.013*	.067`	.048`	.116`	.029`	.032`	0.01	-.081`	.050`	.050`	.035`	.013*	.310`	.062`	.697`	.216`	1					
19	NiceCumEachdNorLn	.131`	.160`	.320`	-.022`	.052`	.012	-.020`	-.031`	0.004	.041`	.092`	.006	.068`	.089`	.317`	.210`	.902`	.248`	1				
20	ProgressIncEachdNor	.001	.022`	.113`	.018**	.085`	.018**	.002	-.015*	-.052`	.021`	.016**	-.01	.01	.177`	.093`	.220`	.160`	.213`	.179`	1			
21	ProgressCumEachdNor	.102`	.195`	.326`	-.062`	-.035`	-.026`	-.032`	-.061`	-.016**	0.005	.049`	-.013*	.043`	.034`	.308`	.065`	.407`	-.003	.406`	.377`	1		
22	TopicIncEachdNor	.072`	.068`	.129`	.047`	.076`	.040`	-0.01	-.024`	-.050`	.031`	.036`	.030`	0.01	.322`	.131`	.377`	.109`	.315`	.089`	.414`	.124`	1	
23	TopicCumEachdNor	.300`	.358`	.449`	.008	-.029`	.035`	-.019**	-.036`	.016**	.021`	.122`	.016**	.061`	.220`	.475`	.248`	.437`	.083`	.355`	.185`	.587`	.289`	1

* Significant at 0.1 level;

** Significant at 0.05 level;

“`” Significant at 0.01 level.

### Variables process

1. Normalization of funding duration
  Since the creator can set the funding duration according to the actual situation of the project itself, the funding duration of each crowdfunding project on platform is different (most between 20–60 days). This kind of data set with different numbers in each period can be treated as unbalanced panel data. The problem of the unbalanced panel is that the statistical software will automatically delete the data according to a certain algorithm. If the discarded data is endogenous, then the estimation will be inconsistent. And if we extract the subset of balanced panel data from unbalanced panel as data set, it will also loss many data and cause the inefficiency of estimation. What's more, no matter the software automatically or we manually extract the sample, it is not random, which will inevitably destroy the randomness of data and reduce the accuracy of the estimation. As we stated above, Kuppuswamy and Bayus [13] and Gu and Zhao [18] deleted many project samples to ensure the balance of the data panel. According to the previous studies [4, 41], the funding period have an important impact on the financing results, so the project selection of 30 days duration could cause the bias of sample.
  To make up for this defect, we will standardize the funding duration for all projects. And take 20 days as the standard length of financing period, the main reasons are as follows:
  (1) Programmatic algorithms can compress projects with an actual financing duration of more than 20 days into a standardized 20-day period. However, for projects with less than 20 days in real duration, it is not possible to stretch them into 20 days (The program code is as follows). For example, if a project with an actual funding duration of 5 days, the funding goal is completed in the next day. So if we need to normalize the actual next day (day 2) into 20 days, we (program code) can't define which standard day it corresponds to. That means we can’t determine which date is the project success date after normalization.
  (2) However, if we make the normalization duration shorter, the interval (including actual days) of normalization days will increase, which is not conducive to observing and analyzing the trend of backers' investment behavior.
  (3) On the contrary, if we make the normalization duration longer, that means more samples will be discarded and the estimation efficiency will reduce.
  (4) Among 927 sample data we collected in this paper, only 64 projects’ funding duration less than 20 days, accounting for 6.9%. Even if these items are eliminated, it will not cause a big impact on the overall samples.
  Combining the above four reasons, and considering that less funding duration is not in line with the original purpose of reward-based crowdfunding. This paper will extract a subset of projects with funding duration longer than 20 days, then compress and normalize them into the length of 20-days.
  Normalized duration is the actual funding days that evenly distributed within 20 days. The pseudo code for normalization is as follows:

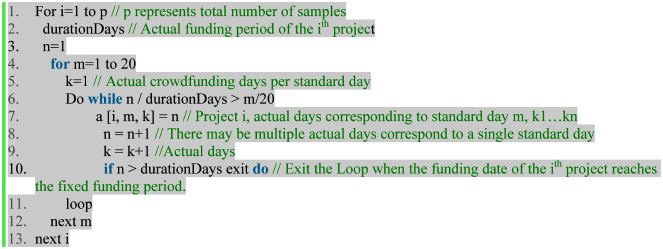
  Take the most commonly used funding duration—30 days as an example. See Table 4 for specific instruction.
2. Dimensional transformation
  In order to reduce the dimensional differences and heteroscedasticity of samples. And to make the data sample approaching to normal distribution, logarithm is used to transfer “Increased funding”, “Cumulative funding”, “Total projects on platform”, “Increased projects on platform”, “Increased backers on platform”, “Cumulative backers on platform”, “Increased backers”, “Cumulative backers”, and several other variables with large range of standard deviation. Because zero and negative value cannot take into logarithm, in order to complete of conversion, we refer to the processing method [42]: for ln(0) use ln (1) to replace and for negative value, use—ln (- a_i_) to transform.

**Table 4:** 30 days duration normalize into 20 days.

Normalized days	1	2	3	4	5	6	7	8	9	10	11	12	13	14	15	16	17	18	19	20
Actual Days	1	23	4	56	7	89	10	11, 12	13	14, 15	16	17, 18	19	20, 21	22	2324	25	2627	28	2930

#### Correlations

From Table 3, there are four pairs of variables shows high correlation. (1) There is a highly positive correlation between the total amount of financing and the total number of projects in all active projects on the platform every day, with a correlation coefficient of 0.959 (p < 0.01). It states that more projects will bring more financing. (2) The correlation coefficient between the cumulative backers and the cumulative funding each day reached 0.806 (p < 0.01). Because the number of backers we used are the actual numbers after deducting the number of “lottery draw” and “charity funding”, each added backer will inevitably lead to an increase to the funding. So, the two variables are highly correlated. (3) There was a significant positive correlation between cumulative focus and popular daily (p < 0.01). (4) The correlation coefficient between Increased focus and popular per day is 0.697 (p < 0.01). These two variables are the performance of the project recognition by the backers, so they have a great correlation. These two variables will be merged in the paper. (5) The cumulative backers on platform is positively and significantly correlated with the cumulative funding on platform, and the correlation coefficient is 0.787(p < 0.01). (6) The increased backers on platform is positively and significantly correlated with the increased funding on platform, and the correlation coefficient is 0.767(p < 0.01). The items (5) and (6) illustrate that more actual supporting backers will bring more financing.

Because the correlation coefficient of these six pairs of variables are high and significant. Therefore, we have classified, merged, or deleted the above six groups of data during model processing, the specific measures are described in the following sections. The correlation coefficients of other variables are below 0.6, so there is no serious multicollinearity problem between other independent variables [43].

In addition, increased projects on platform is not related to the increased funding on platform every day, that indicates new projects won’t bring more financing of the platform, the increased financing of platform is only highly related to the increased actual supporting backers, but has little correlation with other variables.

### Estimation approach

#### Model selection

According to the previous theoretical analysis and hypotheses, as well as the characteristics of the data sets, this paper constructs the panel-data model based on short and balanced panel data, and tests the hypotheses respectively.

Because the pooled regression model ignores the heterogeneity of individuals, and each project of we studies has its own individual characteristics and unobservable heterogeneity, Therefore, like previous scholar [14], we believe that the individual effect model is more suitable in the context of reward-based crowdfunding (the result passed the F test). In addition, selecting an appropriate individual effect model (Fixed Effects Model–FE or Random Effects Model—RE) is a prerequisite for data-panel regression. In FE model, it includes the heterogeneity among the projects. The heterogeneity refers to the differences in certain attributes of projects. Specifically, in reward-based crowdfunding, it can be treated as time invariant information, such as funding goal, video, funding levels, etc. If using FE, it means that the time invariant variables have been controlled. If using RE, all explanatory variables in the regression equation are required to be irrelevant, which means that the model we construct needs to take all influencing factors of the dependent variables into consideration, which is difficult to do in actual situation. That’s why we use FE model in regression model. For the sake of the rigor of the model, Hausman test is carried out for the following regression equations. In addition, because the disturbance items of the same crowdfunding project among different funding dates are not independent distribution in general, and the calculation method of common standard error regards the disturbance items as independent distribution [44], which may lead to inaccurate conclusion, so we use the clustering robust standard error method in the model.

In the below two equations, i means individuals, i.e., the unit of observation; t means time period (Normalization days); Z_*i*_ means variables responsible for unobserved heterogeneity; *u*_*i*_ and *ε*_*it*_ are both disturbance terms.

#### Model equations

Too many variables involved in the explicit and implicit information of project, including moderating effects, which will result in even more variables to be tested. For the convenience of model analysis, the regression equations of explicit and implicit information are constructed according to Hypotheses 1 and 2 respectively. The analysis is as follows:

1. **Equation of Hypotheses 1**. The first half of Eq 1 is all the explicit variables, which can be observed on the project webpage dynamically. The second half Eq 1 is the intersection terms of the dichotomous variable (IsSuccEachNor) with all the explicit variables. By the moderating variable, we can analyze whether the stage of the project will affect the decision-making behavior of backers. Due to Cumulative funding and Cumulative backers are highly correlated, our regression is done separately, but not written in Eq 1. The Hausman test result of Eq 1 shows that *χ*^2^(16) = 52.32, Prob > *χ*^2^ = 0.000. Therefore, the original hypothesis of RE model is strongly rejected, and FE model should be used in Eq 1. And Z_*i*_ should not be included in the actual equation.
2. **Equation of Hypotheses 2**. Eq 2 consists of all the implicit variables and to conserve space the Eq 2 does not include the intersection terms of moderating variable. Owing to the high correlation between total funding on platform and cumulative projects on platform, increased funding on platform and increased projects on platform, total funding on platform and cumulative backers on platform, increased funding on platform and increased backers on platform, 4 pairs of variables. To some extent, it can be understood that the total funding of platform is determined by the number of platform projects and the number of backers on platform, and the increased financing on platform is determined by the increased projects on platform and the increased backers on platform. Therefore, in Eq 2, two variables related to platform financing are removed. Their effect can be proxy by the number of platform projects (total or increase) and the number of platform backers (total or increase). The Hausman test result of Eq 2 shows that *χ*^2^ (10) = 32.32, Prob > *χ*^2^ = 0.000. Therefore, the original hypothesis of RE model is denied, and FE model also should be taken in Eq 2. And Z_*i*_ should not be included in the actual equation.

1. Equation of Hypotheses 1
  $$\begin{array}{l} PledgesRIncNorY_{it}=\beta_{0}+\beta_{1}PledgesRCumNor_{it}+\beta_{2}PledgesCumNorLn_{it}+\beta_{3}BackersCumEachdNorLn_{it}+\beta_{4}DrawNumCumEachdNor_{it}+\beta_{5}DediNumCumEachdNor_{it}\\ +\beta_{6}FocusNiceCumLn_{it}+\beta_{7}ProgressCumEachdNor_{it}+\beta_{8}TopicCumEachdNor_{it}+\beta_{9}IsSuccEachNor_{it}*PledgesRCumNor_{it}+\beta_{10}IsSuccEachNor_{it}*PledgesCumNorLn_{it}\\ +\beta_{11}IsSuccEachNor_{it}*BackersCumEachdNorLn_{it}+\beta_{12}IsSuccEachNor_{it}*DrawNumCumEachdNor_{it}+\beta_{13}IsSuccEachNor_{it}*DediNumCumEachdNor_{it}\\ +\beta_{14}IsSuccEachNor_{it}*FocusNiceCumNorLn+\beta_{15}IsSuccEachNor_{it}*ProgressCumEachdNor_{it}+\beta_{16}IsSuccEachNor_{it}*TopicCumEachdNor_{it}\text{+}\delta {\text{Z}}_{i}+u_{it}+\varepsilon_{it} \end{array}$$ (1)
2. Equation of Hypotheses 2
  $$\begin{array}{l} PledgesRIncNorY_{it}=\beta_{0}+\beta_{1}ActiProjNumEachdNorLn_{it}+\beta_{2}ActiProjNumIncEachdNorLn_{it}+\beta_{3}AllBackersIncEachdNorLn_{it}\\ +\beta_{4}{\begin{array}{l} AllBackersICumEachdNorLn \end{array}}_{it}+\beta_{5}DrawNumIncEachdNor_{it}+\beta_{6}DediNumIncEachdNor_{it}\\ +\beta_{7}BackersIncEachdNorLn_{it}+\beta_{8}FocusNiceIncNorLn_{it}+\beta_{9}ProgressIncEachdNor_{it}+\beta_{10}TopicIncEachdNor_{it}{\text{+Z}}_{i}\text{+}u_{it}+\varepsilon_{it} \end{array}$$ (2)

## Results

In this section, the regression equations designed in prior section will be constructed according to the two hypotheses proposed earlier in this paper. We compared the characteristics of statistical data sets with the regression results and proposed the conclusion in our view. Stata MP 15 software is used for the panel data model test.

### Influence of explicit variables

According to Eq 1, the Increased Funding Rate is used as dependent variable, and 8 variables which could be seen on the project webpage dynamically are treated as independent variables, e.g., Cumulative funding rate, Cumulative funding, Cumulative backers, Cumulative lottery draw, Cumulative dedicators, Cumulative project updates, Cumulative topics, Cumulative focus and popular. The results are shown in column Model1 and column Model2 respectively in Table 5. Since “Cumulative funding” and “Cumulative backers” are highly correlated, the variables PledgesCumNorLn and BackersCumEachdNorLn are in Model1 and Model2 respectively for regression. From the regression results of Model1 and Model2, we can see that the confidence level of all independent variables coefficients is significant under 1%, indicating that the dynamic information on webpage of the project has a significant impact on the decision-making behavior of backers (Increased Funding Rate). As expected in the previous hypotheses, the coefficients of four variables (“Cumulative funding rate”, “Cumulative funding”, “Cumulative backers”, “cumulative dedicators”) in the central route are significantly positive, that means the four hypotheses of H1a, H1b, H1c and H1d are preliminarily validated. Among the variables of peripheral route, the coefficients of five variables of “Cumulative lottery draw”, “Cumulative focus and popular”, “Cumulative project updates”, “Cumulative topics” are all significantly negative, which is also the same with the previous assumptions. Therefore, the five hypotheses of peripheral route, H1d, H1f, H1g, (H1h, H1i), are also preliminarily verified.

**Table 5:** Fixed effect on the explicit signals of projects.

Variable	Model1	Model2	Model3	Model4
PledgesRCumNor	0.0528*** (0.0155)	0.0521*** (0.0156)	0.232*** (0.0233)	0.253*** (0.0233)
PledgesCumNorLn	0.0297*** (0.00547)		0.0202*** (0.00313)	
BackersCumEachdNorLn		0.0429*** (0.0122)		0.0211** (0.01282)
DrawNumCumEachdNor	-0.000780*** (0.000260)	-0.000871*** (0.000243)	-0.000704** (0.000341)	- 0.000668** (0.000301)
DediNumCumEachdNor	0.000554** (0.000239)	0.000511** (0.000239)	0.000238* (0.000140)	0.000230* (0.000138)
FocusNiceCumLn	-0.0493*** (0.0115)	-0.0505*** (0.0127)	-0.0463*** (0.00976)	-0.0451*** (0.0110)
ProgressCumEachdNor	-0.00591* (0.00345)	-0.00603* (0.00352)	-0.00297* (0.00168)	-0.00323* (0.00179)
TopicCumEachdNor	-0.00240*** (0.000632)	-0.00248*** (0.000637)	-0.00232*** (0.000496)	-0.00213*** (0.000519)
IsSuccEachNor_ PledgesRCumNor			-0.184*** (0.0242)	-0.206*** (0.0258)
IsSuccEachNor_ PledgesCumNorLn			-0.019*** (0.00606)	
IsSuccEachNor_ BackersCumEachdNorLn				-0.020*** (0.0122)
IsSuccEachNor_ DrawNumCumEachdNor			-0.0006031* (0.000355)	-0.000521* (0.000309)
IsSuccEachNor_ DediNumCumEachdNor			0.000505** (0.000237)	0.000412* (0.000239)
IsSuccEachNor_ FocusNiceCumNorLn			-0.0398*** (0.0109)	-0.0238** (0.0101)
IsSuccEachNor_ ProgressCumEachdNor			-0.00584 (0.00413)	-0.00579 (0.00409)
IsSuccEachNor_ TopicCumEachdNor			5.66e-05 (0.000511)	-0.000412 (0.000493)
Constant	0.0508 (0.0522)	0.167*** (0.0447)	0.141*** (0.0437)	0.218*** (0.0383)
Observations	17,180	17,180	17,180	17,180
Number of IndividualVar	859	859	859	859
R-squared	0.242	0.230	0.320	0.308

* Significant at 0.1 level;

** Significant at 0.5 level;

*** Significant at 0.01 level;

The numbers in parentheses are standard deviations.

Next is to see how the moderating variable (IsSuccEachNor) changes regression results. Similarly, the variables PledgesCumNorLn and BackersCumEachdNorLn cannot simultaneously enter the Eq 1 because of the high correlation between the “Cumulative backers” and “Cumulative Funding”, which showed in Model3 and Model4 in Table 5, respectively. From the results, the moderating variable does not change the direction and significance of main effect coefficient in Eq 1. However, the moderating variable (crowdfunding results) significantly affect the dependent variable through five variables: “Cumulative funding rate”, “Cumulative funding / Cumulative backers”, “Cumulative lottery draw”, “Cumulative dedicators”, “Cumulative focus and popular”. We will analyze the regression results one by one in the following part.

1. Moderating effect on “Cumulative funding rate”
  Regardless of Model3 or Model4, the partial regression coefficients *β*_9_ for interactive term IsSuccEachNor*PledgesRCumNor of “Crowdfunding Result” and “Cumulative funding rate” are significantly negative (-0.184, -0.206), verified the hypothesis H1j. This indicates that when the project succeeds, the main effect of “Cumulative funding rate” weakens, and after adding the moderating effect, the slope of “Cumulative funding rate” turns flat (Model3:0.232–0.184 = 0.048; Model4:0.047). It can be concluded that: (1) although there is herding effect in the whole financing period, the intensity of backers’ investment behavior weakens gradually. This conclusion is also in line with the other scholars' argument [33, 45] that the herding effect on backers' behavior exists at the beginning of funding period, then the marginal effect will decline or disappear. (2) It also shows that the Bystander Effect is more obvious after the project reaches funding goal, because with the success of the project, the attraction of the project to potential backers will gradually decrease, and they are tending to fund other projects that have not been successful yet.
2. Moderating effect on “Cumulative funding / Cumulative backers”
  From the Model 3 in Table 5, it shows that after adding the interactive item to the model, the result of partial regression coefficient *β*_10_ is significantly negative (- 0.019, 0.006), which further verified the hypothesis H1k proposed in previous section. Although the main regression coefficient of “Cumulative funding” is still significantly positive, the value decreases compared with the coefficient *β*_2_ in Model 1, indicating that the positive effect of “Cumulative funding” on backers' investment behavior turns weak when the project is unsuccessful. However, the partial regression coefficient *β*_10_ of the interaction item decreased more obviously (0.0202–0.019 = 0.0012) after the project success, which denotes that under the moderating effect, the influence of "Cumulative funding" on backers' decision-making behavior is significantly weakened. It also shows that the herding effect appears a weakening trend.
3. Moderating effect on “Cumulative dedicators”
  Both in Model3 and Model4, the partial regression coefficients *β*_13_ of IsSuccEachNor*DediNumCumEachdNor, an interactive term of “Crowdfunding results” and “Cumulative dedicators”, are significantly positive (Model3: 0.000505, Model4: 0.000412), and in the same direction with the main effect. This is different from the hypothesis H1m proposed previous in this paper. This indicates that the “crowdfunding results” has an enhanced moderating effect on the “Cumulative dedicators”. And this effect will be more obvious after the project success. So, the hypothesis H1m is denied.
4. Moderating effect on “Cumulative lottery draw” and “Cumulative focus and popular”
  From Model3 and Model4 in Table 5, it shows that the moderating effect of “crowdfunding results” on these two variables is consistent with the previous assumptions, and both are significantly negative. Therefore, the hypotheses of H1n, H1q, H1r are verified preliminarily.
5. Moderating effect on “Cumulative project updates” and “Cumulative topics”
  No matter in Model3 or Model4, the interactive terms of IsSuccEachNor*ProgressCumEachdNor and IsSuccEachNor*TopicCumEachdNor are not significant, which indicates that the moderator will not affect the backers’ investment behavior by “Cumulative project updates” and “Cumulative topics”. And the coefficients in the main effects of these two variables *β*_7_ and *β*_8_, relative to the corresponding coefficients in Model1 and Model2, have the same direction of influence on backers behavior, but relatively moderate. Therefore, hypotheses H1o, H1p in the previous paper failed to pass the test.

From the regression results in Table 5, especially after using “crowdfunding results” as moderating variable, the goodness of fit R^2^ of Model 3 and Model 4 is higher than that of Model 1 and Model 2, which shows that the model with moderating variable is more suitable to interpret the dependent variable. In addition, based on the above moderating effect analysis and results of dynamic regression model of the longitudinal data panel, most assumptions in Hypothesis1 are verified. It denotes that the dynamic explicit information that display on the webpage have a significant impact on backers' investment decision-making behavior.

### Influence of implicit variables

After the regression according to Eq 2, the “Total projects on platform”, “Increased projects on platform”, “Cumulative backers on platform”, “Increased lottery draw”, “Increased dedicators”, “Increased backers”, “Increased focus and popular”, “Increased updates”, “Increased topics”, 9 implicit variables in total have significant influence on the dependent variable. Only the regression result of “Increased backers on platform” variable is not significant.

Among the six variables in the central route, the coefficients of “Total projects on platform” and “Increased projects on platform” are significant and negative, which preliminarily verifies the hypotheses H2c and H2d in this paper and illustrates the existence of “Kickstarter fatigue”; The coefficients of “Cumulative backers on platform”, “Increased backers”, “Increased dedicators” three explanatory variables are significant and positive, which also are in line with the hypotheses proposed above. The preliminary validation of the hypotheses H2a, H2e, H2f are proved. Only the coefficient (*β*_3_) of “Increased backers on platform” is not significant (p > 0.1), indicating that this explanatory variable has no significant effect on backers' investment decision-making behavior. This may be due to the fact that the increased backers on the platform will largely converge on good quality projects or near-successful projects. For most of the ordinary projects on the platform, that will not have much attraction for the Increased the backers, so it has no significant impact on the increased funding rate of their own project. Therefore, the Hypothesis H2b has not been validated.

The coefficients in the peripheral route, such as “Increment lottery draw”, “Increased focus and popular”, “Increased project updates” and “Increased topics” are all significantly negative, which conform to the hypotheses put forward earlier section: H2r, H2s, H2t, H2u, H2v. This states that variables with pro-social attributes have a negative effect on the backers’ investment behavior, which is also consistent with the regression results of explicit variables, indicating that the more interactions, the less support backers because of the Bystander Effect.

In addition, we also take the “crowdfunding results” as a moderating variable to construct the FE panel data model, and regress the implicit dynamic variables in the central and peripheral route to verify the previous hypotheses H2i-H2v. The regression results show that except for the "Increased backers", the moderating variable has no effect on other implicit variables (p > 0.1), and the fitting goodness of model (R^2^ = 0.092) is smaller than the model without the moderating effect (R^2^ = 0.211). Moreover, the whole regression model of the Eq 2 with the moderating variables has not passed the F-test, which indicates that the Eq 2 is not meaningful as a whole. That shows the “crowdfunding results” cannot moderate the information which is not easy or can’t be observed. This paper will no longer discuss it and won’t list the regression results, due to the length of the paper.

**Fig 3:**
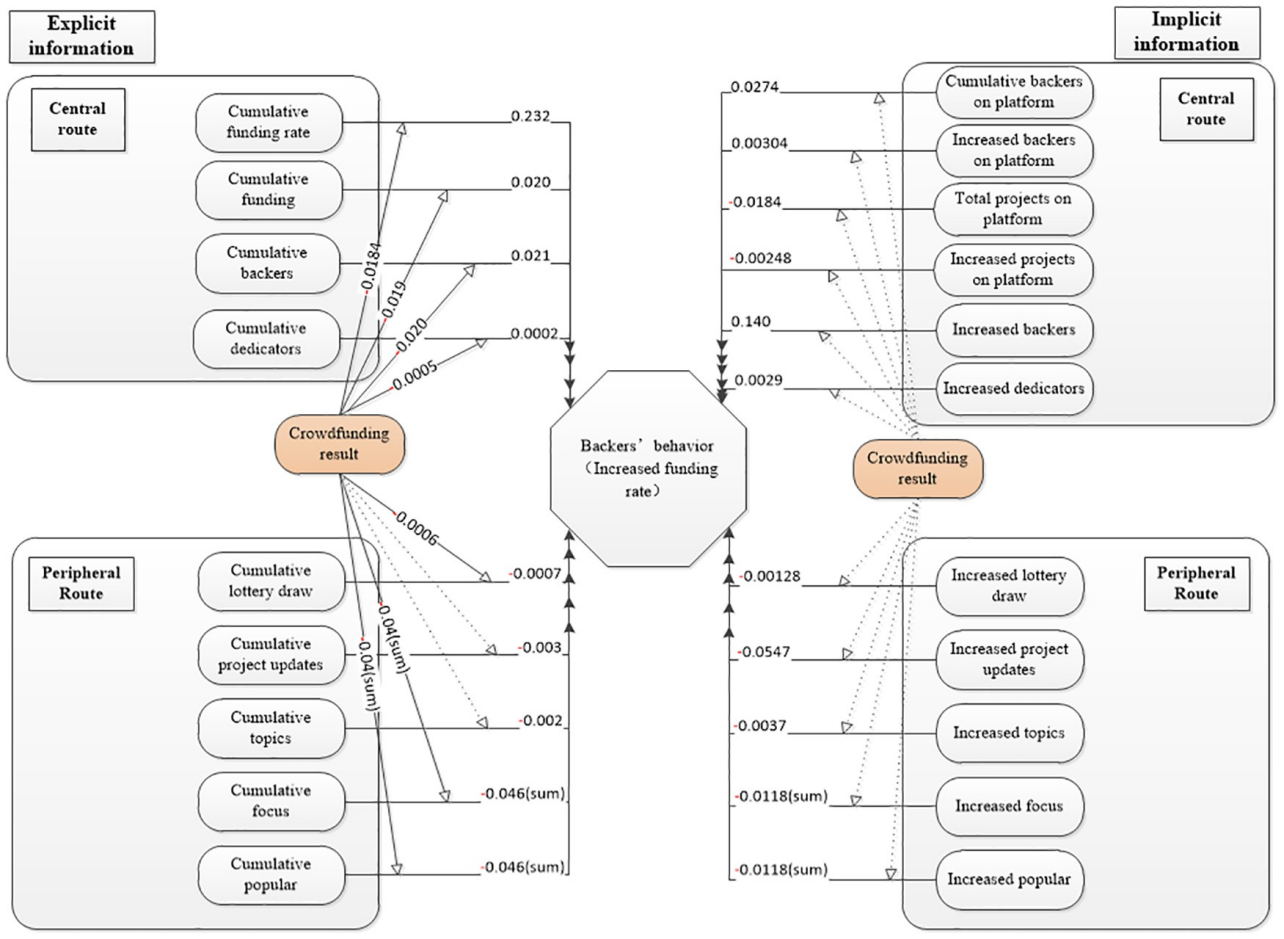
Explicit and implicit variables structure of analysis results. The arrow with dotted line means that there is no moderating effect on the point variable, i.e., P > 0.05.

Through the above regression results and analysis of the explicit and implicit variables in central and periphery route of crowdfunding projects. The Hypotheses 1 and 2 are verified, and to some extent, the results indicate that the existence of herding effect during the whole funding period but its intensity has a weakening tendency. After adding the moderating variable of “crowdfunding results”, we analyzed the change of the coefficient in the model before and after the success of the project, and got the influence factors of explicit and implicit information on backers' investment behavior in the project financing cycle. Considering the regression results and Fig 1, we give the structure equation figure as Fig 3.

### Robustness checks

As explained above, in addition to the Increased Funding Rate, we can also use Increased Funding as the independent variable. The Increased Funding Rate focuses on establishing the direct relationship between the backers’ investment behavior and the funding goal of project, while the Increased Funding focuses more on actual investment amount and willingness of supporting backers. Therefore, in order to ensure the robustness of the results of the previous regression, this section uses Increased Funding as the dependent variable, and again tests the FE data panel model of Eqs 1 and 2 with the independent variables unchanged. The results are as follows:

#### Robustness checks of influence of explicit variables

Regression results for Eq 1 are shown in Table 6, compared with the results in Table 5, the direction of the coefficients of each variable did not change, except that the significance of each individual variable was little higher than before, but remained within the range of statistical confidence. It illustrates that the previous Hypotheses 1 are validated and the explicit variables in Eq 1 passes the robustness test.

**Table 6:** Fixed effect on the explicit signals of projects using PledgesIncrNorYLn as dependent variable.

Variable	Model1	Model2	Model3	Model4
PledgesRCumNor	0.174** (0.0866)	0.201** (0.0980)	1.910*** (0.329)	3.248*** (0.373)
PledgesCumNorLn	0.491*** (0.0380)		0.374*** (0.0393)	
BackersCumEachdNorLn		0.498*** (0.0873)		0.151* (0.091)
DrawNumCumEachdNor	-0.0034* (0.00205)	-0.00329* (0.00198)	-0.00741*** (0.00225)	-0.00836*** (0.00246)
DediNumCumEachdNor	0.00093624* (0.000564)	-0.000879* (0.000529)	0.000909*** (0.000320)	0.000765** (0.000323)
FocusNiceCumLn	-0.819*** (0.0935)	-0.755*** (0.103)	-0.807*** (0.0921)	-0.723*** (0.0984)
ProgressCumEachdNor	-0.165*** 0.174**	-0.162*** (0.0284)	-0.178*** (0.0334)	-0.175*** (0.0332)
TopicCumEachdNor	(0.0866) 0.491***	-0.0167*** (0.00556)	-0.0234*** (0.00673)	-0.0203*** (0.00680)
IsSuccEachNor_ PledgesRCumNor			-1.746*** (0.328)	-3.070*** (0.367)
IsSuccEachNor_ PledgesCumNorLn			-0.0899** (0.0458)	
IsSuccEachNor_ BackersCumEachdNorLn				-0.428*** (0.0983)
IsSuccEachNor_ DrawNumCumEachdNor			-0.00761*** (0.00259)	-0.00914*** (0.00265)
IsSuccEachNor_ DediNumCumEachdNor			0.00217*** (0.000552)	0.00145*** (0.000498)
IsSuccEachNor_ FocusNiceCumNorLn			-0.072376* (0.0436)	-0.1464* (0.0882)
IsSuccEachNor_ ProgressCumEachdNor			0.0402 (0.0410)	0.0395 (0.0406)
IsSuccEachNor_ TopicCumEachdNor			0.00554 (0.00618)	0.000596 (0.00629)
Constant	3.734*** (0.430)	5.943*** (0.406)	4.322*** (0.427)	6.622*** (0.398)
Observations	17,180	17,180	17,180	17,180
Number of IndividualVar	859	859	859	859
R-squared	0.047	0.038	0.051	0.050

* Significant at 0.01 level;

** Significant at 0.05 level;

*** Significant at 0.001 level;

The numbers in parentheses are standard deviations.

#### Robustness checks of influence of implicit variables

With other conditions unchanged (independent variables and moderating variable), using Increased Funding as the dependent variable, FE model regressed again according to the Eq 2. Similarly, “crowdfunding results” used as the moderating variable to regress with the central and peripheral route of implicit variables, and it is shown that there is no moderated effect (significance index p > 0.1) of “crowdfunding results” with all implicit variables, and the whole regression model has not passed the F-test, so “crowdfunding results” has no moderated effect on implicit information variables. Except for the test results of the moderated variable, the results of main effect variables listed in Table 7, compared with the results in Table 8, we can conclude that the coefficient direction of each variable has not changed, and the result of the “Increased backers on platform” variable is still not significant. Thus, the previous Hypotheses 2 are proofed, and the implicit variables in Eq 2 passed the robustness test.

**Table 7:** FE model on the implicit signals of projects using PledgesIncrNorYLn as dependent variable.

Variable	Model	Variable	Model
ActiProjNumEachdNorLn	-0.333*** (0.0660)	DediNumIncEachdNor	0.0107*** (0.00157)
ActiProjNumIncEachdNorLn	-0.0174* (0.0105)	BackersIncEachdNorLn	2.115*** (0.0497)
AllBackersIncEachdNorLn	0.00145 (0.0144)	FocusNiceIncNorLn	-0.0428* (0.0258)
AllBackersICumEachdNorLn	0.407*** (0.0766)	ProgressIncEachdNor	-0.0873* (0.0526)
DrawNumIncEachdNor	-0.0260*** (0.00356)	TopicIncEachdNor	-0.0225** (0.00943)
Constant	3.947*** (0.605)	Observations	17,180
		Number of IndividualVar	859

* Significant at 0.1 level;

** Significant at 0.05 level;

*** Significant at 0.01 level;

The numbers in parentheses are standard deviations.

**Table 8:** Fixed effect on the implicit signals of projects.

Variable	Model	Variable	Model
ActiProjNumEachdNorLn	-0.0184** (0.00884)	DediNumIncEachdNor	0.0029* (0.00161)
ActiProjNumIncEachdNorLn	-0.00248* (0.00145)	BackersIncEachdNorLn	0.140*** (0.0282)
AllBackersIncEachdNorLn	0.00304 (0.00414)	FocusNiceIncNorLn	-0.0118* (0.00633)
AllBackersICumEachdNorLn	0.0274*** (0.00993)	ProgressIncEachdNor	-0.0547* (0.0297)
DrawNumIncEachdNor	-0.00128* (0.000657)	TopicIncEachdNor	-0.0037*** (0.00108)
Constant	-0.243*** (0.0919)	Observations	17,180
		Number of IndividualVar	859

* Significant at 0.1 level;

** Significant at 0.05 level;

*** Significant at 0.01 level;

The numbers in parentheses are standard deviations.

From the robustness test results of Eqs 1 and 2, all the Hypotheses pass the robustness test in general, but the test results show that the goodness of fit of each model is not as good as when using the Increased Funding Rate as the dependent variable.

## Conclusion and discussion

### Conclusion

This paper focuses on the backers’ investment decision-making behavior of reward-based crowdfunding. Based on ELM model, take explicit and implicit dynamic information variables in the central and peripheral route, and use “crowdfunding results” as moderating variable in FE panel-data model. According to the regression results and statistical analysis, our finding that there are many variables of the central route will have a positive and significant impact on backers' investment behavior, while most variables in the periphery route have a negative and significant impact on backers' investment. The funding status (crowdfunding results) has a significant moderating effect on the explicit variables of the project, and the effect is mostly negative; but the funding status has no significant moderating effect on the implicit information variables. Finally, while analyzing the influence of the above factors on backers' behavior, the regression results also verify that there is herding effect in crowdfunding, but its intensity will gradually weaken under the goal gradient effect. Therefore, herding effects only highlight in early project funding period well before the target goal draws near [10]. Moreover, herding effect and goal gradient effect have no interaction with each other.

### Contributions and managerial implications

Our research highlights the importance of dynamic information displays on the webpage whether will impact on the backers’ investment behavior. From the results, we suggest that the creator can improve the crowdfunding project from the following aspects.

1. Creators should make full use of the effects at the beginning of the project. Before the herding effect is weakened and the diffusion of responsibility effect is enhanced, they should strive for as much financing as possible to reach the goal gradient effect of the project as soon as possible. For example, a funder can pledge early funding from friends and family, or set “early bird” in the project funding level to give the supporter extra reward the first few hours.
2. Setting a reasonable funding goal plays a key role in reward-based crowdfunding project success. Fig 2 illustrates that there are 13.3% projects end up less than 5% of the funding goal and 9.3% of the projects end up at the 105% of their funding goal. Nearly 90% of projects end up funding 2.5 times of their funding goal. Even on the Kickstarter platform, 45% of successful projects will ultimately raise less than 110% of their funding goal, and 66% of failed projects will ultimately raise less than 10% of the funding goal [10]. A rational funding goal can trigger the goal gradient effect as early as possible. The creator need refer to the past projects of the same category and decide a reasonable funding goal according to the quality information that the project itself can convey to the backers.
3. Adjusting the real-time information on the webpage, enhance the positive factors and weaken the negative factors which could impact on the backers’ investment behavior. Fixed information is not suitable for attracting backers during the different funding stages. Jensen et al. [46] points out that the long-term display of the words like “we only need your 500 dollars” or “each cent counts” would significantly reduce the funding efficiency. Therefore, we recommend that creator should adjust the dynamic display information on the webpage at any time according to progress of the project. For instance, when approaching the funding goal of a project, adding helping words in the webpage that can enhance potential backers' perceived impact, and make them feel their pledge at this point can bring key help to the creators, and so on. In addition, creators should use real time information on the webpage to reduce the negative impact (e.g., pro-social factors) of project on backers' decision-making behavior. For different crowdfunding platforms, creators should design their displayed information according to each platform rules.

For crowdfunding platform, they can also take corresponding platform strategies according to the backers' investment behavior. For example, in the beginning, in order to help trigger the goal gradient effect early, extensive propaganda and display should be done before the project launch; In the middle of the funding period, platform could set a series of category, e.g., items such as high-focus projects or high-innovation projects, to attract potential backers’ attention; At the end of project funding period, we advise that projects should be treated differently according to the current funding rate. Projects with more than 80% funding rate should be displayed more to help the 6.1% of the creators to complete their funding goal (according to Fig 2, 6.1% of the projects terminate at 85% of funding goal). Moreover, in term of Limited Attention Theory, if the funding rate has not exceeded 50% in the near-end stage of the project (from Fig 2, about 35% of projects), there is no need for the platform to waste valuable display place on the platform to promote them.

Our finding contributes to ELM literature. Although the theory has been applied to reward-based crowdfunding context, it is first time applied in dynamic implicit information factors. Moreover, unlike prior studies of panel data model, we improved the method of variables selection, saved large amount of data set, which will increase model accuracy and reduce estimation error. It offers new insights into the dynamic implicit information of reward-based crowdfunding behavior of backers, and more broadly.

### Limitations and further research directions

#### Limitations

1. Although we improved the econometric approach and the data set included 28878 samples, the time span is only 4 months, lacking the measurement and analysis of annual data. The data set cannot observe whether seasonal changes have an impact on backers' investment behavior.
2. The data sets based only on Chinese reward-based crowdfunding platform as the research object. Although JD platform leads reward-based crowdfunding platform in China and could represent the overall trend of Chinese crowdfunding, the data set used in this paper lacks the research on other countries’ crowdfunding platforms in the world.

#### Further research direction

Although we analyzed the impact of dynamic explicit and implicit information on backers' investment decision-making behavior, we do not build a prediction model of project results. In order to extend the research field, based on the dynamic panel data in this paper, prediction models can also be constructed according to the situation of each project's daily data. For ongoing projects, the dynamic prediction model (e.g., BP or MLP Neural Network Model) can be used to provide investment decision reference for potential backers, as well as give real-time management for platform.

## Supporting information

S1 Code (TXT)

S1 Data (XLSX)
